# Overexpression of homeodomain-interacting protein kinase 2 (HIPK2) attenuates sepsis-mediated liver injury by restoring autophagy

**DOI:** 10.1038/s41419-018-0838-9

**Published:** 2018-08-28

**Authors:** Zhengyu Jiang, Lulong Bo, Yan Meng, Chen Wang, Tianxing Chen, Changli Wang, Xiya Yu, Xiaoming Deng

**Affiliations:** 1 Faculty of Anesthesiology, Changhai Hospital, Second Military Medical University, Shanghai, 200433 China; 2 Department of Cell Biology, School of Basic Medicine, Second Military Medical University, Shanghai, 200433 China; 3 School of Life Science, Nanjing University, 210023 Nanjing, Jiangsu Province China; 4 State Key Laboratory of Pharmaceutical Biotechnology, Nanjing University, 210023 Nanjing, Jiangsu Province China

## Abstract

Sepsis is the leading cause of death in intensive care units worldwide. Autophagy has recently been shown to protect against sepsis-induced liver injury. Here, we investigated the roles of homeodomain-interacting protein kinase 2 (HIPK2) in the molecular mechanism of sepsis-induced liver injury. HIPK2 expression was reduced in sepsis-induced liver injury, and HIPK2 overexpression increased the survival rate and improved caecal ligation and puncture (CLP)-induced liver injury by reducing serum and liver aspartate transaminase (AST), alanine transaminase (ALT), and alkaline phosphatase (ALP) levels in mice with sepsis. HIPK2 overexpression significantly decreased CLP-induced release of inflammatory cytokines into the serum and attenuated oxidative stress-associated indicators in mice with CLP-induced liver injury, whereas HIPK2 knockdown produced the opposite results, suggesting that HIPK2 is a negative regulator of sepsis. Furthermore, HIPK2 overexpression inhibited lipopolysaccharide (LPS)-induced apoptosis of primary hepatocytes, increased the autophagic flux, and restored both autophagosome and autolysosome formation in the livers of CLP-induced mice by suppressing calpain signalling. Importantly, HIPK2 overexpression reduced the elevated cytosolic Ca^2+^ concentration in LPS-treated primary hepatocytes by interacting with calpain 1 and calmodulin. Finally, several anti-inflammatory drugs, including resveratrol, aspirin, vitamin E and ursolic acid, significantly increased the levels of the HIPK2 mRNA and protein by modulating promoter activity and the 3′-UTR stability of the HIPK2 gene. In conclusion, HIPK2 overexpression may improve sepsis-induced liver injury by restoring autophagy and thus might be a promising target for the clinical treatment of sepsis.

## Introduction

Sepsis has been considered the leading cause of death in intensive care units worldwide^1^. Survivors of sepsis showed higher readmission rates and more severe pathological courses than patients without sepsis^1^. The 2016 definition of sepsis (known as Sepsis-3) has acknowledged the importance of organ dysfunction/failure. Based on the sequential organ failure assessment score, organ failure has been considered an essential component required to diagnose sepsis^2^. The liver plays an essential role in immunological homeostasis and metabolism^3^. Liver dysfunction has been considered an early indicator of a poor prognosis of sepsis^4^, and strategies designed to restore liver function result in a better prognosis and outcome in patients with sepsis^5^. Initially, sepsis-induced liver dysfunction is induced by multiple pathogenic factors^6^, including lipopolysaccharide (LPS), inflammatory factors, or pathogens^5,7–9^. The roles of complex reactions and substances, such as reactive oxygen species (ROS), nitrogen species (RNS), inflammation, and apoptosis^10–12^, as well as alterations in hepatocytes, such as autophagy and apoptosis, in the progression of sepsis have been widely investigated^8,9,13,14^.

Autophagy is a conserved and catabolic process in which proteins and organelles are isolated by a double-membrane vesicle and targeted to the lysosome for proteolytic degradation^15^. Upregulation of autophagy attenuates the inflammatory response and improves the survival rate by reducing organ dysfunction^16^, whereas inhibition of autophagy has been reported to result in increased mortality by exacerbating injury induced by multiple factors and depleting immune cells in patients with sepsis^17,18^. Thus, autophagy might be a promising target for managing sepsis. Recent studies using a caecal ligation and puncture model (CLP) to induce sepsis have observed a significant increase in autophagy in the mouse liver that was identical to clinical patients^19,20^, and the activation of autophagy may be enhanced by genipin and growth arrest and DNA damage inducible protein 34 (GADD34), which exert protective effects against sepsis^13,14^. Importantly, inhibition of mTOR exerts protective effects against acute kidney injury in mice with endotoxaemia^21^. A loss of Ca^2+^ homeostasis affects the activity of the Ca^2+^-dependent cysteine protease calpain, leading to organ injury^22^. The calpain system suppresses the progression of autophagy by directly cleaving Atg proteins throughout the autophagy course^23,24^. Furthermore, activities and levels of calpain proteins are increased in CLP-induced sepsis models, indicating that calpains may inhibit autophagy in sepsis^25^. In this study, we focused on the autophagic flux and its related signalling pathways in CLP-induced sepsis.

Homeodomain-interacting protein kinase 2 (HIPK2) is a serine/threonine kinase that is mainly located in the nucleus^26^. HIPK2 is a potential tumour suppressor, as it promotes apoptosis in response to chemotherapeutic drugs and radiation, mainly by phosphorylating p53^27,28^. HIPK2 also protects cells against genome instability by enhancing DNA damage repair signalling^29^. Nevertheless, HIPK2 can also support tumour progression. HIPK2 is significantly overexpressed in cervical cancer^30^, pilocytic astrocytoma^31^, and ovarian and prostate tumours compared with normal samples^31,32^, and it is also overexpressed in aggressive meningiomas compared with benign meningiomas^33^. Furthermore, HIPK2 functionally interacts with NRF2^34^, a transcription factor involved in protecting the liver and autophagy^35–40^. However, the precise effects of HIPK2 on autophagy and sepsis-induced liver injury remain unclear. As CLP animal model is regarded as a better choice to simulate sepsis development in the clinic^19,20,41^, we investigated the molecular mechanisms underlying the protective effect of HIPK2 on CLP-induced sepsis, devoting our attention to the mechanisms regulating autophagy.

## Materials and methods

### Animals and adenovirus preparation

Eight-week-old C57 BL/6 male mice weighing ~ 20.8 g each were used in this study. All mice were purchased from the Experimental Animal Centre of the Second Military Medical University (Shanghai, China). Animals were housed in a specific pathogen-free room in cages with sawdust bedding at a temperature of ~ 25 °C, a relative humidity of ~ 50%, 12 h light/day, and free access to water and food. All procedures performed on the animals used in this study were approved by the Animal Care and Protection Committee of the Second Military Medical University. The authors confirm that the animals received human care, and all animal experiments were performed in accordance with the relevant guidelines and regulations.

Recombinant adenoviruses expressing mouse HIPK2 (Ad-HIPK2) and an shRNA targeting HIPK2 (Ad-shHIPK2) were generated using the pAd-Easy system (Invitrogen, California, USA). The Ad-vector or Ad-NC was used as the negative control. We inserted a mouse albumin promoter in the adenovirus system to exclusively express the adenovirus in the liver. The obtained viruses were diluted with phosphate-buffered saline (PBS) and injected at a concentration of 2 × 10^9^ plaque-forming units into each mouse via the tail vein. All other chemicals were purchased from Sigma.

### Experimental design and CLP model of sepsis

In this study, sepsis was induced by performing a CLP as reported previously^42^. Mice were randomly divided into several groups: the control (sham) group and the CLP-induced group were pretreated with Ad-vector or Ad-NC, Ad-HIPK2, or Ad-shHIPK2 (30 mice for each group), and 2 × 10^9^ plaque-forming units were injected into each mouse every 12 days. The CLP surgery was performed on the fifth day after the adenovirus injection. Bafilomycin A (10 mg/kg per day), chloroquine (50 mg/kg per day) or rapamycin (1 mg/kg per day) was administered 2 h prior to CLP. The liver and serum were obtained 16 h after CLP-induced sepsis for further studies. During the CLP process, the mice were anaesthetized with sevoflurane, and then a midline abdominal incision was performed. The caecum was ligated at one-half of the distal terminal and was perforated to trigger bacterial peritonitis. Next, the abdominal wall was sutured in two layers and subcutaneously injected with 1 mL of a 0.9% sodium chloride solution for fluid resuscitation. A laparotomy and bowel operation without ligation and perforation were performed on the mice in the control (sham) group. The chemicals used to establish the model were purchased from Sigma.

### Analyses of liver function

As indicators of liver function, serum, and liver levels of glutathione (GSH), alanine transaminase (ALT), alkaline phosphatase (ALP), and aspartate transaminase (AST) were analysed by employing biochemical kits from Nanjing Jiancheng Bioengineering Institute (Nanjing, China).

### Analyses of H_2_O_2_ and O^.−^_2_ production and ROS levels in the mouse liver

Hepatic levels of O^.−^_2_ were measured using the chemiluminescence method^43^. First, the mouse liver tissues were weighed and homogenized in pH 7.4 lysis buffer containing 10 mM ethylenediaminetetraacetic acid (EDTA) and 20 mM 4-(2-hydroxyethyl)-1-piperazineethanesulfonic acid (HEPES). Samples were centrifuged at 1000 g for 10 min. Then, an aliquot of each sample was incubated with Krebs-HEPES buffer, pH 7.4, containing 5 mM lucigenin (Sigma, Shanghai, China) for ~ 2 min at 37 °C. Next, light emission data were obtained using a M200 PRO multifunctional microplate reader (TECAN, Switzerland), and the results are reported as the mean light units min/mg protein. Levels of O^.−^_2_ were measured by adding superoxide dismutase (SOD) (350 U/mL) to the medium according to the manufacturer’s instruction (Nanjing Jiancheng Bioengineering Institute, Nanjing, China). In addition, liver tissues were homogenized in normal saline, and the samples were treated with an equal volume of cold methanol for 60 min in the 4 °C icebox. Then, samples were centrifuged for at 10,000 g for 30 min, and we obtained the supernatant to measure H_2_O_2_ levels using biochemical kits (Nanjing Jiancheng Bioengineering Institute, Nanjing, China). Protein concentrations were measured using the Bradford method, and bovine serum albumin (BSA) was employed as the standard.

### ELISAs for the determination of IL-1β, TNF-α, and IL-16 levels

The weighed liver tissues were added to a cold PBS buffer (pH 7.0) containing 0.002% sodium azide, 0.1 mg/mL soybean trypsin inhibitor, 2 mM phenylmethylsulfonyl fluoride, 10 nM EDTA, and 1.0 mg/mL BSA. The tissues were homogenated, and samples were then incubated for 2 h in a 4 °C refrigerator. For further assays, the supernatants were collected by centrifugation at 12,000 g for 10 min. IL-1β, TNF-α, and IL-16 levels in the supernatant of serum and liver samples were measured using ELISA kits (R&D Systems, Shanghai, China).

### Haematoxylin–eosin (H & E) and immunohistochemical (IHC) staining

Mouse liver tissues were fixed with 10% formalin, and the fixed specimens were processed into paraffin blocks, sectioned (5 μm), and stained with H & E for the histological analysis using standard protocols^44^.

Liver sections were subjected to dewaxing with xylene and dehydration in a graded ethanol series. Sections were incubated with 3% H_2_O_2_ for 10 min to block the activity of endogenous peroxidases. Then, sections were heated to 100 °C in 0.1 M citrate buffer (pH 6.0) for 30 min for antigen retrieval. These liver tissues were incubated with anti-LC3-II and anti-LAMP-2 antibodies overnight at 4 °C. Next, the tissues were incubated with horseradish peroxidase-conjugated anti-rabbit or anti-mouse IgG secondary antibodies (Envision kit, Dako, Denmark) according to the manufacturer’s instructions. Finally, these sections were stained with haematoxylin. The stained sections were photographed with a Carl Zeiss microscope (Axio Observer A1, Jena, Germany). The semi-quantitative analysis was performed by two investigators who were blinded to the experimental design; these investigators measured the optical density using ImageJ software^45^.

### Reverse transcription-polymerase chain reaction (RT-PCR)

RT-PCR and quantitative real-time PCR (Q-PCR) were performed as previously described^46^. Total RNA was extracted from liver tissues using TRIZOL reagent from Thermo Fisher Scientific (Waltham, MA, USA). The complementary DNA templates were obtained by reverse transcription in a 10 μL reaction containing 1 μg of total RNA, oligo (dT) primers, and a reverse transcription premix. Q-PCR was performed with the SYBR green PCR system in an ABI 7500 thermal cycler (Thermo Fisher Scientific, Waltham, MA, USA). The SYBR green reagents were also purchased from Thermo Fisher Scientific. The following cycling conditions were used: 95 °C for 3 min followed by 40 cycles of denaturation at 95 °C for 10 s, annealing at 60 °C for 5 s, and extension at 72 °C for 10 s. The mRNA levels were normalized to the level of the β-actin mRNA, which was used as an internal control. The primers were: glyceraldehyde 3-phosphate dehydrogenase (GAPDH), sense, 5′-CATTCAAGACCGGACAGAGG-3′, antisense, 5′-ACATACTGCACACCAGCATCACC-3′; HIPK2, sense, 5′-CCAGGC CTGCTTGCTCAG-3′, antisense, 5′-TGTACAGATGTGTGGGTGGC-3′. Finally, we determined the relative mRNA levels using the 2^−ΔΔCt^ method, and GAPDH was used as an internal control.

### Immunoblot analysis

Liver tissues or cells were lysed in radioimmunoprecipitation assay buffer (1 mM EDTA, pH 8.0, 50 mM Tris-HCl, pH 8.0, 2% sodium dodecyl sulfate, and 5 mM dithiothreitol), and protein concentrations were determined using a bicinchoninic acid assay (Beyotime Inc., Shanghai, China). The total protein samples (~ 30 μg) were separated on an sodium dodecyl sulfate polyacrylamide gel electrophoresis gel, transferred to PVDF (polyvinylidene fluoride) membranes (Invitrogen, California, USA), and blocked with 5% non-fat dry milk in PBST (phosphate-buffered saline with Tween), pH 7.5. Membranes were immunoblotted with primary antibodies for 4 h or overnight at 4 °C and then incubated with horseradish peroxidase-conjugated secondary antibodies. The anti-HIPK2 (#5091), anti-NRF2 (#12721), anti-NQO1 (#62262), anti-GAPDH (#5174), anti-p62 (#88588), Beclin-1 (#3738), Atg3 (#3415), Atg7 (#2631), LAMP-2 (#49067), Rab7 (#2094), cathepsin B (#3373), anti-mTOR (#2972), anti-phospho-mTOR (Ser2448) (#2971), anti-4E-BP1 (#9452), anti-phospho-4E-BP1 (Ser65) (174A9) (#9456), anti-p70 S6 kinase (#9202), and anti-phosphoplus p70 S6 kinase (Thr389) (#9234) antibodies were purchased from Cell Signalling Technology (MA, USA). The anti-calpain 1 (NBP1-88205), anti-calpain 2 (NBP1-15675), anti-Atg5 (NB110-53818), anti-LC3-II (NBP1-19167), anti-calmodulin, and other antibodies were purchased from Novus Biologicals (CO, USA). The horseradish peroxidase-conjected secondary antibodies were all purchased from Cell Signalling Technology (MA, USA). Protein bands were detected with an enhanced chemiluminescence kit (Pierce, Rockford, USA). The corresponding semi-quantitative analysis was performed by measuring the optical density using ImageJ software, and GAPDH was used as an internal control.

### Isolation of primary hepatocytes

Mouse primary hepatocytes were isolated as previously described^47^. C57 BL/6 mice were anaesthetized with 1% pelltobarbitalum natricum (Amresco, USA). The mice underwent surgical dissection, the hepatic portal veins were washed to remove the blood, and a collagenase (Sigma-Aldrich, USA) perfusion was performed. The livers were immediately moved to a sterile 10-cm cell culture dishes for mincing before the hepatocytes were dispersed by aspiration with a large-bore pipette; the hepatocytes were then filtered through a 70-μm membrane (Millipore, USA) to remove tissue debris. After two washes with cold Dulbecco’s Modified Eagle’s Medium (DMEM) and centrifugation at 50 g for 4 min at 4 °C, the isolated hepatocytes were seeded in 6-cm dishes containing DMEM supplemented with 10% fetal bovine serum (FBS) (both from Invitrogen, USA) at a density of 1 × 10^7^ cells/dish. The medium was changed 6 h after seeding.

### Determination of primary hepatocyte apoptosis using flow cytometry

Apoptotic cells were analysed using an annexin V-fluorescein isothiocyanate (FITC)/propidium iodide (PI) apoptosis detection kit (Beyotime, Shanghai, China). The primary hepatocytes were first isolated from the mouse liver using a previously described procedure^48^. Then, cells were seeded in plates and cultured overnight in DMEM supplemented with 10% FBS. Hepatocytes were transfected with Ad-HIPK2 or Ad-vector (2 × 10^3^ plaque-forming units per well) for 16 h, followed by an incubation with 0.5 μg/mL LPS for another 12 h. Cells were harvested and centrifuged at 250 g for 5 min at 4 °C and resuspended in cold binding buffer (10 mM HEPES buffer, pH 7.4, 5 mM KCl, 150 mM NaCl, 1.8 mM CaCl_2_, and 1 mM MgCl_2_). Next, hepatocytes were stained with FITC-labelled 25 ng/mL FITC-labelled annexin V in the dark for 40 min, followed by an incubation with 50 ng/mL propidium iodide for 5 min. Samples were analysed with a Becton Dickinson flow cytometer (USA). The results were analysed with flow cytometry software. In the present study, apoptotic cells were stained with annexin V but not PI. Hepatocytes stained with both annexin V and PI were considered necrotic or late apoptotic cells.

### Measurement of cytosolic Ca^2+^ levels by flow cytometry

Cytosolic Ca^2+^ levels were measured by flow cytometry using previously described procedures^49^. First, the isolated primary hepatocytes were seeded in a six-well plate and grown to 50% confluence before the application of the indicated treatment. After 12 h of the adenovirus infection, cells were treated with 1 μg/mL LPS for another 12 h. Then, samples were washed and stained with 5 μM Fluo-4 AM according to the manufacturer’s instructions (Invitrogen, CA, USA). The intercellular Ca^2+^ levels were measured in the FL1 channel using a flow cytometer.

### Immunoprecipitation

The immunoprecipitation was conducted as previously described^50^. In brief, primary hepatocytes were incubated with LPS (0.5 μg/mL) for 12 h. Then, cells were collected and lysed in a buffer containing 20 mM PIPES, pH 6.8, 1% Triton X-100, 150 mM NaCl, 150 mM sucrose, 0.2% sodium deoxycholate, 500 μM EDTA, and protease inhibitors using a mortar for 5 min on ice. After centrifugation, supernatants were diluted to 2 μg/mL in dilution buffer containing 20 mM PIPES, pH 6.8, 1% Triton X-100, 150 mM NaCl, 150 mM sucrose, 2.5 mM MgCl_2_, and 2.5 mM MnCl_2_. Primary antibody-conjugated protein A beads were incubated with the lysates for 2 h at 4 °C before washing with dilution buffer. The subsequent immunoblot analysis was conducted using the abovementioned methods. The chemicals were purchased from Sigma.

### Luciferase reporter assay

The predicted 3′-UTR sequence (295 bp) of HIPK2 was cloned from the genomic DNA of primary hepatocytes, and then the sequence was inserted into the pmir-GLO empty luciferase reporter vector (Promega, WI, USA). The predicted promoter sequence (−300 bp to + 10 bp) of HIPK2 was inserted into the pGL-3 basic luciferase reporter vector from Promega. For further studies, primary hepatocytes were co-transfected with the internal control vector pmir-GLO (Promega) and pmir-GLO-HIPK2 3′-UTR using the Lipofectamine 2000 reagent (Thermo Fisher Scientific) for 20 h, followed by an incubation with the indicated chemicals for 10 h. Similarly, hepatocytes were co-transfected with the empty vector pGL-3 basic (Promega) and the pGL-3 vector containing the HIPK2 promoter using the Lipofectamine 2000 reagent for 20 h; the pRL vector was used as an internal control. Cells were then incubated with the indicated chemicals for 10 h. The luciferase reporter activities were determined using the Dual-Glo luciferase assay system from Promega. The drugs and concentrations used in the present study were as follows: 5 μM resveratrol, 30 μM aspirin, 10 μM vitamin E, and 15 μM ursolic acid. The compounds were purchased from MedChemExpress NJ, USA).

### Statistical analysis

All data are presented as means ± standard deviations. Data were analysed using Student’ s *t* tests (comparisons between two groups) or a one-way analysis of variance test (comparisons between multiple group) followed by a post hoc analysis using Tukey’s honestly significant difference test with SPSS 17.0 software (SPSS, Inc., Chicago, IL, USA). Differences were considered statistically significant with a *p* value < 0.05.

## Results

### Expression of HIPK2 in the livers of CLP-induced mice

**Fig. 1:**
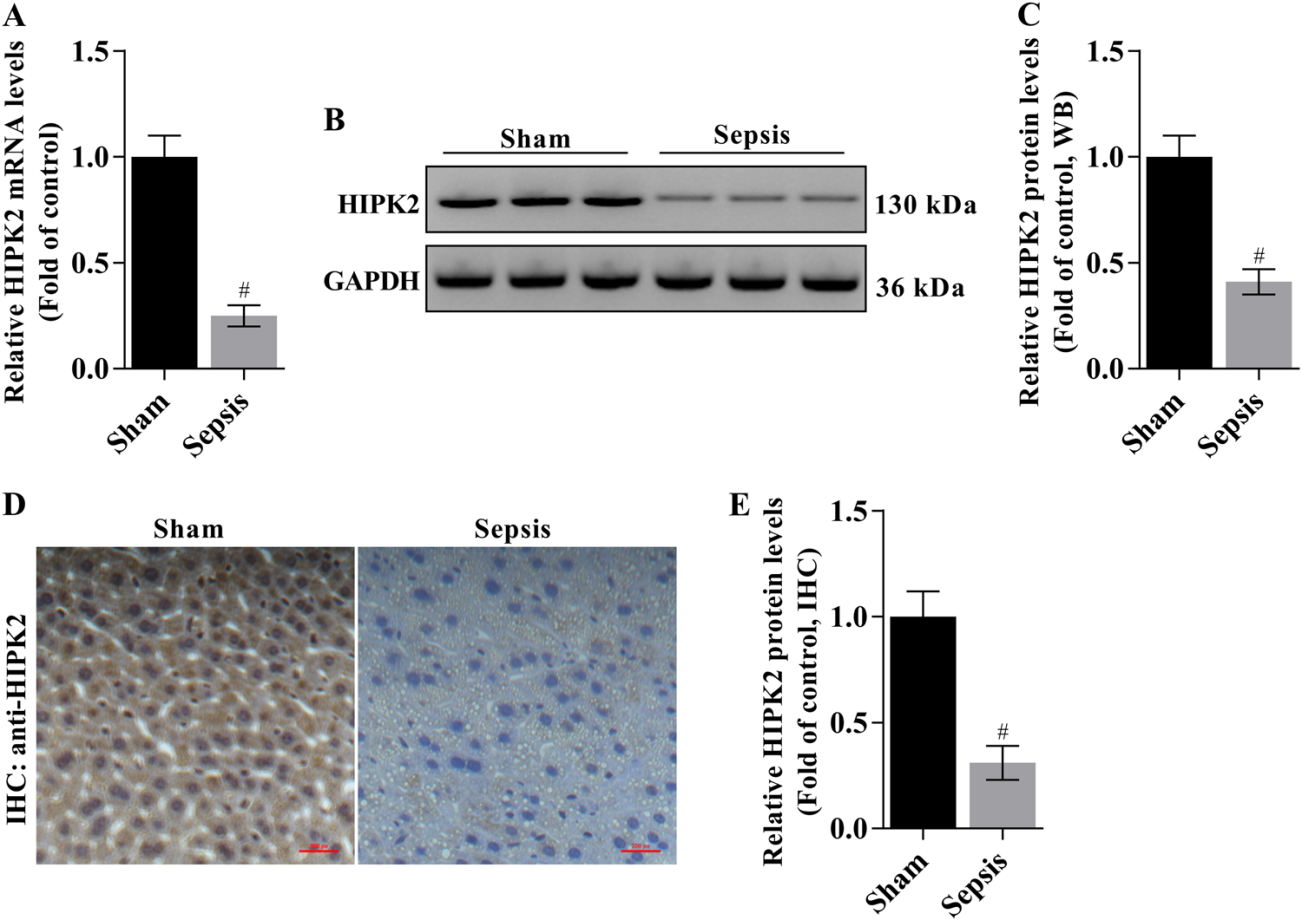
HIPK2 is expressed at lower levels in liver tissues from mice with CLP-induced liver injury. **a** Analysis of levels of the HIPK2 mRNA by Q-PCR; GAPDH was used as an internal control. The data are presented as means ± SEM (*n* = 3), #*p* < 0.05 compared with the sham group. **b**, **c** Western blot analysis of levels of the HIPK2 protein (**b**) and the results of the corresponding semi-quantitative analysis of levels of the HIPK2 protein based on the optical density measured using ImageJ software (**c**); the data are presented as means ± SEM and are representative of three separate experiments, #*p* < 0.05 compared with the sham group. **d**, **e** Images of IHC staining for the HIPK2 protein (**d**) and the results of the corresponding semi-quantitative analysis of levels of the HIPK2 protein based on the optical density measured using ImageJ software (**e**). The data are presented as means ± SEM (*n* = 3), #*p* < 0.05 compared with the sham group. The liver and serum samples were obtained from mice 16 h after the CLP surgery

We analysed hepatic HIPK2 expression to initially examine the alterations in HIPK2 expression during sepsis. Lower levels of the HIPK2 mRNA were observed in the livers of CLP-induced mice, as analysed by Q-PCR (Fig. 1a). Similarly, a lower level of the HIPK2 protein was detected in liver tissues from CLP-induced mice than in the control group (sham), as determined by western blotting and IHC (Fig. 1b–e).

### HIPK2 overexpression increases the survival rate and improves CLP-induced liver injury in mice

**Fig. 2:**
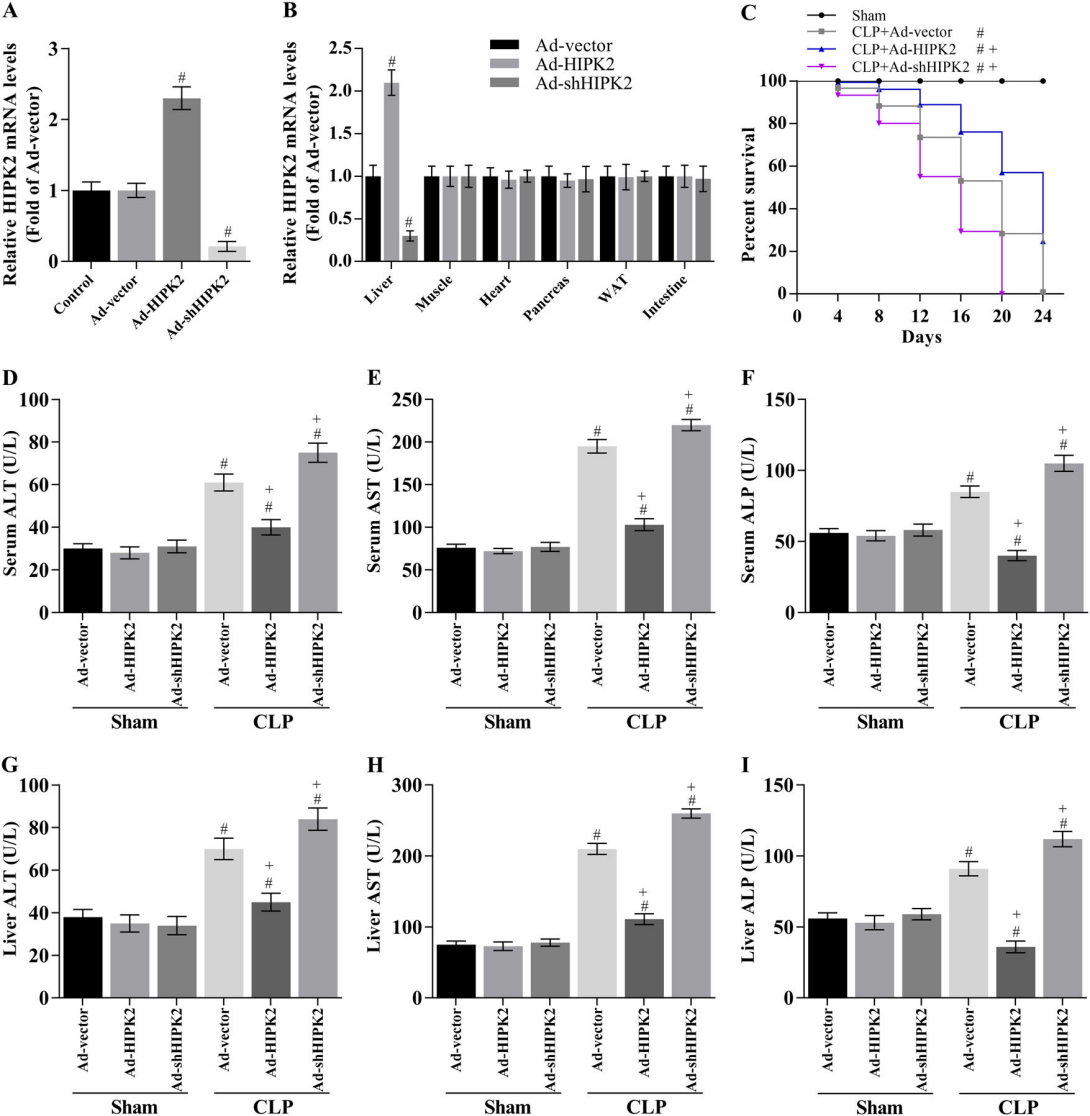
Effects of HIPK2 on sepsis-induced lethality and production of liver injury-related factors in CLP-induced mice. **a** HIPK2 was overexpressed following the injection of Ad-HIPK2 and HIPK2 was knocked down following the injection of Ad-shHIPK2, and hepatic HIPK2 expression was analysed by Q-PCR; the data are presented as means ± SEM (*n* = 3), #*p* < 0.05 compared with the Ad-vector group. **b** The expression of HIPK2 in several different tissues was analysed by Q-PCR; the data are presented as means ± SEM (*n* = 3), #*p* < 0.05 compared with the Ad-vector group. **c** Effect of HIPK2 on the survival of mice with CLP-induced sepsis; the data are presented as means ± SEM (*n* = 30 mice per group), #*p* < 0.05 compared with the sham plus Ad-vector group, + *p* < 0.05 compared with the CLP plus Ad-vector group. In this section, the CLP was performed on the fifth day after the adenovirus injection, and the liver and serum were obtained 16 h after CLP-induced sepsis. **d**, **e**, **f** Effect of HIPK2 on serum AST, ALT, and ALP concentrations, as determined by ELISAs; the data are presented as means ± SEM (*n* = 30 mice per group), #*p* < 0.05 compared with the sham plus Ad-vector group, + *p* < 0.05 compared with the CLP plus Ad-vector group. **g**, **h**, **i** Effect of HIPK2 on liver AST, ALT, and ALP levels, as analysed by ELISAs; the data are presented as means ± SEM (*n* = 30 mice per group), #*p* < 0.05 compared with the sham plus Ad-vector group, + *p* < 0.05 compared with the CLP plus Ad-vector group

Next, we overexpressed HIPK2 to investigate possible hepatic alterations. The Ad-HIPK2 and Ad-shHIPK2 adenoviruses successfully altered HIPK2 expression in the liver but not in other organs (Fig. 2a, b). Based on this premise, the 16-day survival rate was 16.7% (5 of 30 mice) in the CLP-induced sepsis group, 60.0% (18 of 30 mice) in the Ad-HIPK2-injected sepsis group, 6.67% (2 of 30 mice) in the Ad-shHIPK2-injected sepsis group (Fig. 2c) and 100% in the control group (30 mice). Compared with that in the sepsis group, the 16-day survival rate of the Ad-HIPK2-treated sepsis group was improved (*p* < 0.05) (Fig. 2c), indicating that the pretreatment of mice with Ad-HIPK2 before CLP significantly decreased lethality compared to that in animals with CLP-induced sepsis. However, knockdown of HIPK2 by Ad-shHIPK2 may increase lethality.

Serum and liver levels of the liver-specific enzymes AST, ALT, and ALP were determined to further analyse the extent of CLP-induced liver dysfunction. Higher AST, ALT, and ALP levels were observed in septic mice than in the sham (normal) group, and Ad-HIPK2 significantly decreased AST, ALT, and ALP levels in the serum and liver of septic mice (Fig. 2d–i). In contrast, knockdown of HIPK2 (Ad-shHIPK2) increased the AST, ALT, and ALP levels in the serum and liver of septic mice (Fig. 2d–i). Combined with the survival rate in Fig. 2c, the data suggest that HIPK2 exerts a protective role in sepsis and sepsis-related acute liver injury that might be related to its ability to decrease the hepatic ALT, AST, and ALP contents.

### HIPK2 overexpression reduces the CLP-induced release of inflammatory cytokines into the serum and the levels of oxidative stress-associated indicators in CLP-induced mice

**Fig. 3:**
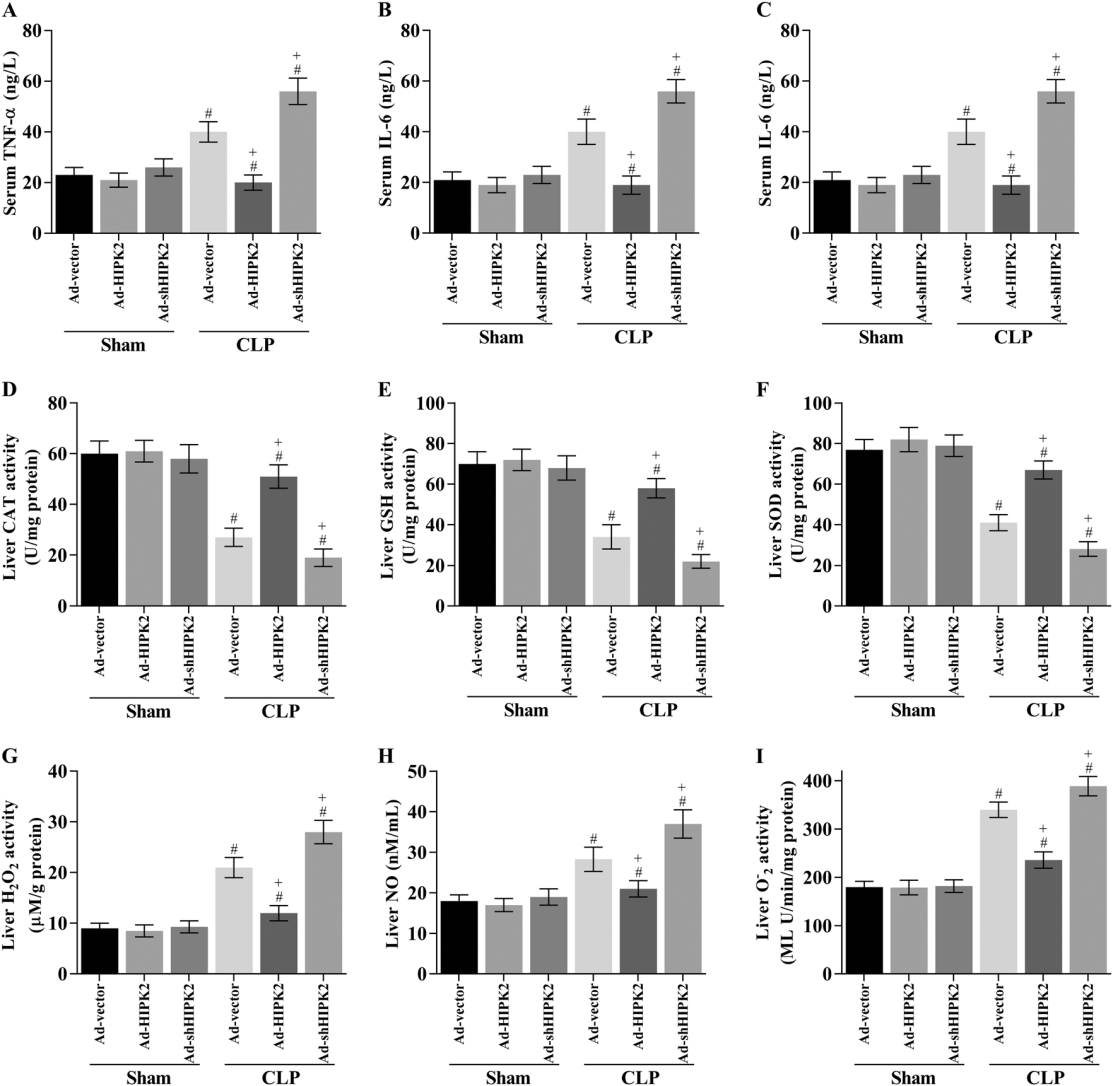
Effects of HIPK2 on the levels of inflammatory cytokines and oxidative stress-related markers in CLP-induced mice. The CLP surgery was performed on the fifth day after the adenovirus injection and the liver and serum were obtained 16 h after CLP-induced sepsis. **a**, **b**, **c** Serum TNF-α, IL-1β and IL-6 concentrations were determined using ELISAs; data are presented as means ± SEM (*n* = 30 mice per group), #*p* < 0.05 compared with the sham plus Ad-vector group, + *p* < 0.05 compared with the CLP plus Ad-vector group. **d–i** Liver CAT (**d**), GSH (**e**), SOD (**f**), H_2_O_2_ (**g**), NO (**h**), and O^.−^_2_ (**i**) concentrations were measured using ELISAs; data are presented as means ± SEM (*n* = 30 mice per group), #*p* < 0.05 compared with the sham plus Ad-vector group, + *p* < 0.05 compared with the CLP plus Ad-vector group

We measured the serum concentrations of TNF-α, IL-6, and IL-1β; analysed the levels of antioxidants such as SOD, GSH, and CAT; and detected the levels of H_2_O_2_, O^.−^_2_, and NO in the liver to determine the effects of HIPK2 on inflammation and oxidative stress in the livers of LPS-induced mice. Serum TNF-α, IL-6, and IL-1β levels in the CLP group were 40.6 ± 3.1 ng/L, 38.5 ± 2.8 ng/L and 7.7 ± 1.9 ng/L, respectively (Fig. 3a–c). HIPK2 overexpression significantly decreased serum TNF-α (19.2 ± 2.4 ng/L), IL-6 (18.7 ± 2.1 ng/L), and IL-1β (3.4 ± 1.2 ng/L) levels by ~ 52.7%, 51.4%, and 72.6%, respectively. However, HIPK2 knockdown decreased the serum concentrations of TNF-α (54.5 ± 3.6 ng/L), IL-6 (56.1 ± 4.0 ng/L), and IL-1β (8.3 ± 1.5 ng/L) by ~ 25.8%, 34.2%, and 7.8%, respectively (Fig. 3a–c).

On the other hand, the activities of SOD, CAT, and GSH were significantly decreased in mice with CLP-induced sepsis. As expected, HIPK2 overexpression evidently restored CAT (Fig. 3d), GSH (Fig. 3e), and SOD activities (Fig. 3f). Furthermore, hepatic H_2_O_2_, O^.−^_2_, and NO levels were analysed in mice. The H_2_O_2_, O^.−^_2_, and NO levels were elevated in the livers of CLP-induced mice, and HIPK2 overexpression significantly inhibited H_2_O_2_, O^.−^_2_, and NO production in the livers of CLP model mice (Fig. 3g–i). In contrast, HIPK2 knockdown obviously decreased the SOD, CAT, and GSH activities and increased the release of H_2_O_2_, O^.−^_2_, and NO (Fig. 3d–i). Interestingly, the expression of the antioxidant transcription factor NRF2 and its target gene NQO1 increased after HIPK2 was overexpressed in septic mice (Supporting Fig. 1A and 1B), providing a potential explanation for the mechanism by which HIPK2 overexpression reduces oxidative stress. Thus, we reasonably assumed that reductions in ROS production and oxidative stress might be involved in the protective effects of HIPK2 on the liver in CLP-induced mice.

### HIPK2 overexpression increases the autophagic flux in the livers of CLP-induced mice and inhibits LPS-induced apoptosis of primary hepatocytes

**Fig. 4:**
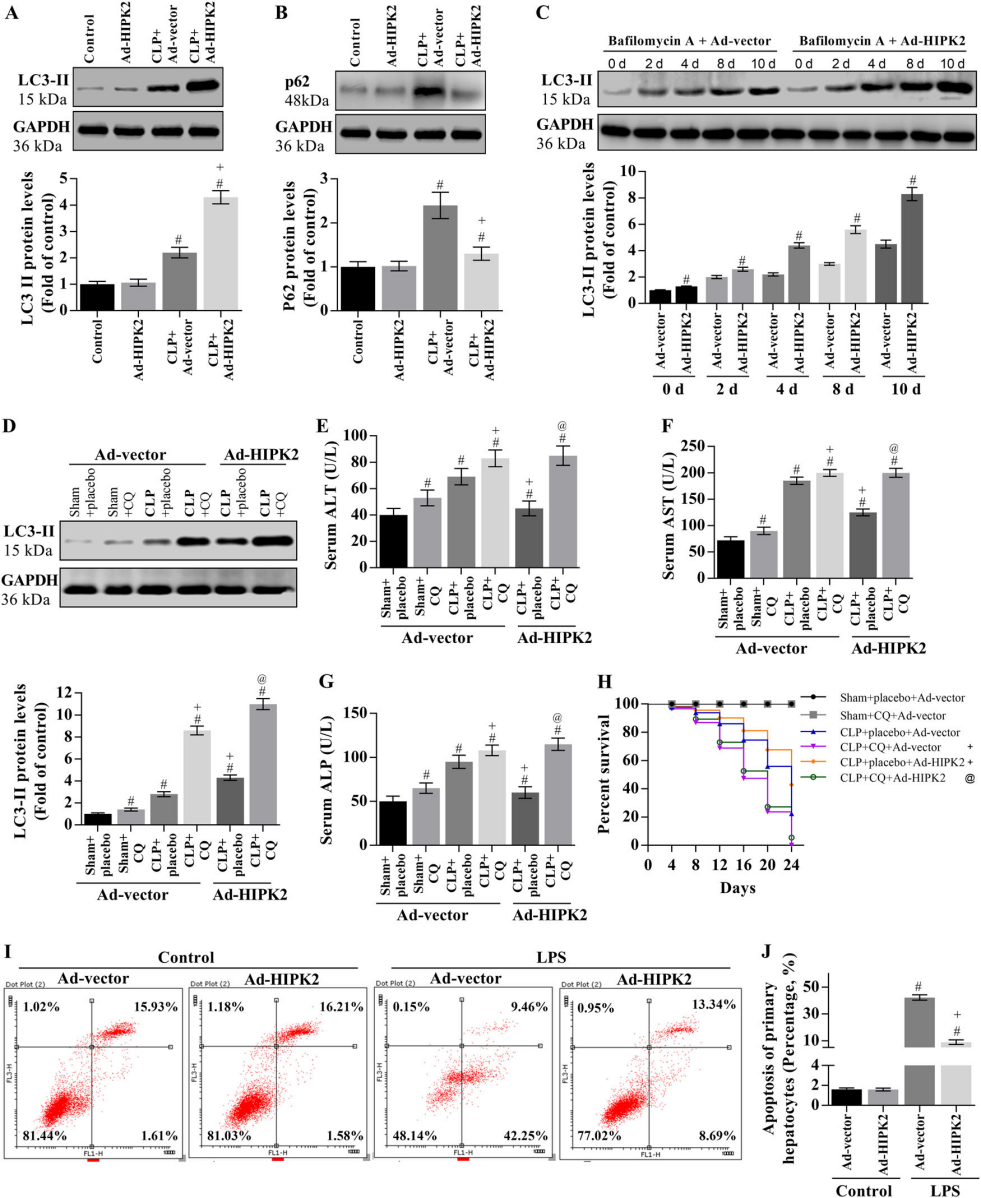
HIPK2 overexpression increases the autophagic flux in mice with CLP-induced liver injury and inhibits the LPS-induced apoptosis of primary hepatocytes. **a**, **b** Effects of HIPK2 on levels of the LC3-II and p62 proteins in the liver; the data are presented as means ± SEM and are representative of three separate experiments, #*p* < 0.05 compared with the control group and + *p* < 0.05 compared with the CLP plus Ad-vector group. **c** Effects of Ad-HIPK2 on hepatic LC3-II expression in of the group treated with bafilomycin A (0.2 μM); the data are presented as means ± SEM and are representative of three separate experiments, #*p* < 0.05 compared with the Ad-vector (control) group at the indicated time point. **d** Effect of chloroquine (CQ, 50 mg/kg per day) on levels of the LC3-II protein in the liver; the data are presented as means ± SEM and are representative of three separate experiments, #*p* < 0.05 compared with the sham plus Ad-vector group in the absence of CQ, + *p* < 0.05 compared with the CLP plus Ad-vector group in the absence of CQ, and @*p* < 0.05 compared with the CLP plus Ad-HIPK2 group in the absence of CQ. **e**, **f**, **g** Effect of chloroquine (CQ) on serum concentrations of AST, ALT, and ALP in the HIPK2-overexpressing liver tissues, as determined by ELISAs; the data are presented as means ± SEM (*n* = 30 mice per group), #*p* < 0.05 compared with the sham plus Ad-vector group in the absence of CQ, + *p* < 0.05 compared with the CLP plus Ad-vector group in the absence of CQ, and @*p* < 0.05 compared with the CLP plus Ad-HIPK2 group in the absence of CQ. **h** Effect of chloroquine (CQ) on the sepsis-induced lethality of HIPK2-overexpressing mice (*n* = 30 mice per group); + *p* < 0.05 compared with the CLP plus Ad-vector group in the absence of CQ and @*p* < 0.05 compared with the CLP plus Ad-HIPK2 group in the absence of CQ. In this section, CLP (*n* = 30) was performed on the fifth day after the adenovirus injection, and CQ (50 mg/kg) was administered 1 h prior to CLP. **i** Effect of HIPK2 overexpression on LPS (0.5 μg/mL)-induced apoptosis of primary hepatocytes, as analysed by flow cytometry. **j** Percentage of apoptotic cells induced by LPS; data are presented as means ± SEM and are representative of three separate experiments, #*p* < 0.05 compared with the Ad-vector group, + *p* < 0.05 compared with the LPS plus Ad-vector group

We analysed the levels of the LC3-II and p62 proteins to investigate the effect of HIPK2 on autophagic flux in the livers of CLP-induced mice. In the livers of CLP-induced mice, the levels of the LC3-II and p62 proteins were significantly increased by ~ 2.1- and 2.5-fold, respectively, compared with those in the control group on the fourth day after CLP (Fig. 4a, b). HIPK2 overexpression increased LC3-II expression (Fig. 4a, b). Bafilomycin A was used to detect the autophagic flux in mice. Bafilomycin A significantly increased the levels of the LC3-II protein. Furthermore, Ad-HIPK2 may enhance the bafilomycin A-induced accumulation of LC3-II (Fig. 4c). Then, we used chloroquine (an autophagic flux inhibitor) to determine the contribution of autophagy to liver protection. Chloroquine increased LC3-II expression in the CLP model (Fig. 4d). Moreover, chloroquine reversed the HIPK2-mediated decrease in mortality and liver injury in CLP mice and increased ALT, AST, and ALP activities in CLP mice compared with those observed following the administration of Ad-HIPK2 alone (Fig. 4e–h).

We also investigated the effect of HIPK2 on the apoptosis of primary hepatocytes. HIPK2 overexpression significantly attenuated the LPS-induced apoptosis of primary hepatocytes (Fig. 4i, j).

### HIPK2 overexpression restores the formation of autophagosomes and autolysosomes in CLP-induced mice

**Fig. 5:**
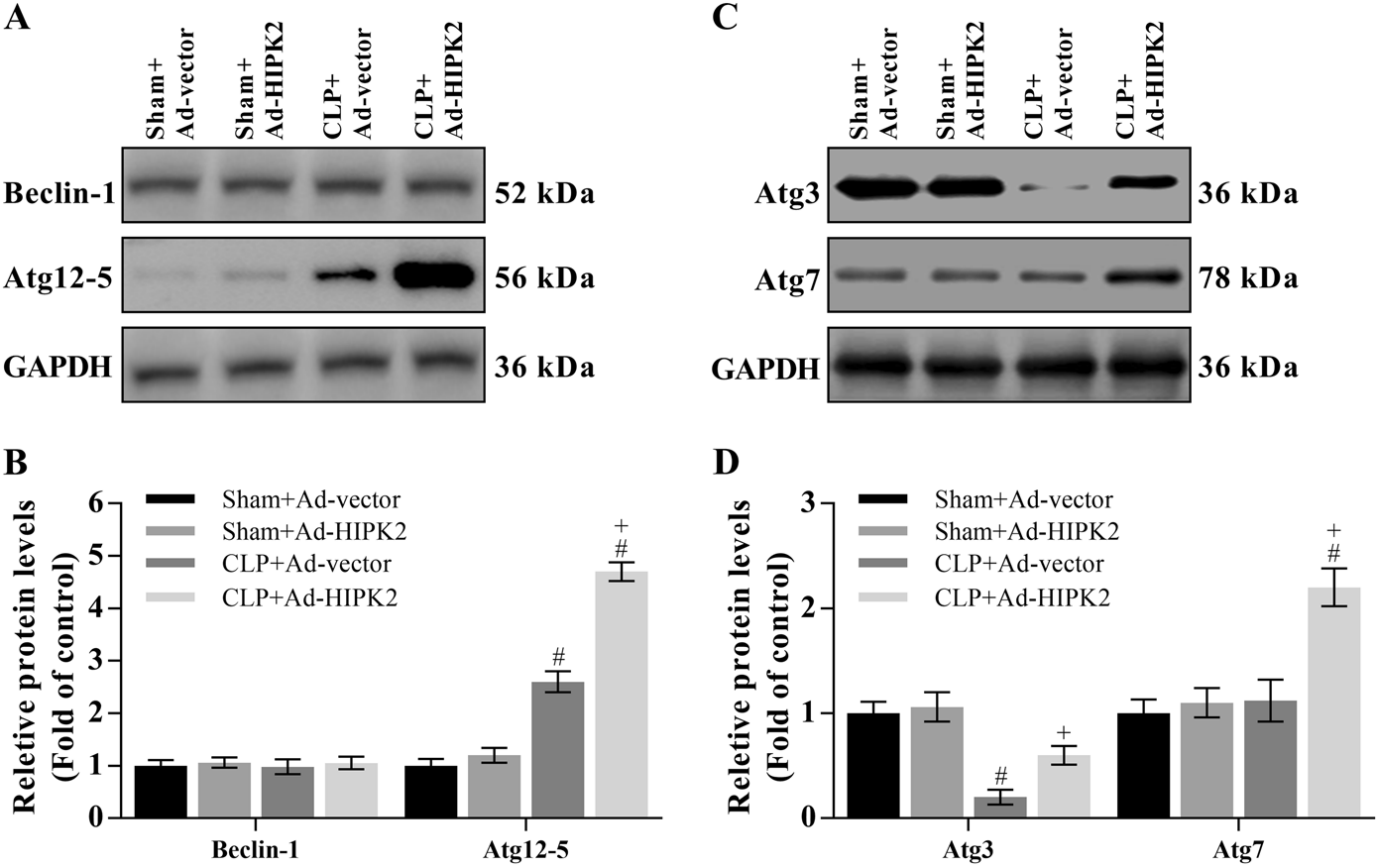
HIPK2 overexpression restores autophagosome formation in CLP-induced mice. **a** Effect of HIPK2 overexpression on Beclin-1 and Atg12-5 levels, as analysed by western blotting; CLP (*n* = 30) was performed on the fourth day after the adenovirus injection. Data are presented as means ± SEM and are representative of three separate experiments; #*p* < 0.05 compared with the sham plus Ad-vector group (control), + *p* < 0.05 compared with the CLP plus Ad-vector group. **b** Effect of HIPK2 overexpression on Atg3 and Atg7 levels, as analysed by western blotting. Data are presented as means ± SEM and are representative of three separate experiments, #*p* < 0.05 compared with the sham plus Ad-vector group, + *p* < 0.05 compared with the CLP plus Ad-vector group

The mature autophagosome is formed by the Atg12-5-16 L1 complex, Beclin-1, complex and LC3-II. In the present study, the level of the Atg12-5 protein was increased by 1.5-fold in CLP-induced mice compared with that in the control mice, and this increase was enhanced by HIPK2 overexpression (Fig. 5a, b). However, Beclin-1 expression was not changed by sepsis or HIPK2 overexpression (Fig. 5a, b). Moreover, we analysed the levels of markers of LC3 lipidation, including Atg3 and Atg7. Atg3 expression was reduced by 76.4% compared with that in the control group, and this reduction was reversed by HIPK2 overexpression (Fig. 5c, d). Furthermore, Atg7 expression was not changed by CLP-induced sepsis, while the levels of this protein were increased upon HIPK2 overexpression (Fig. 5c, d).

**Fig. 6:**
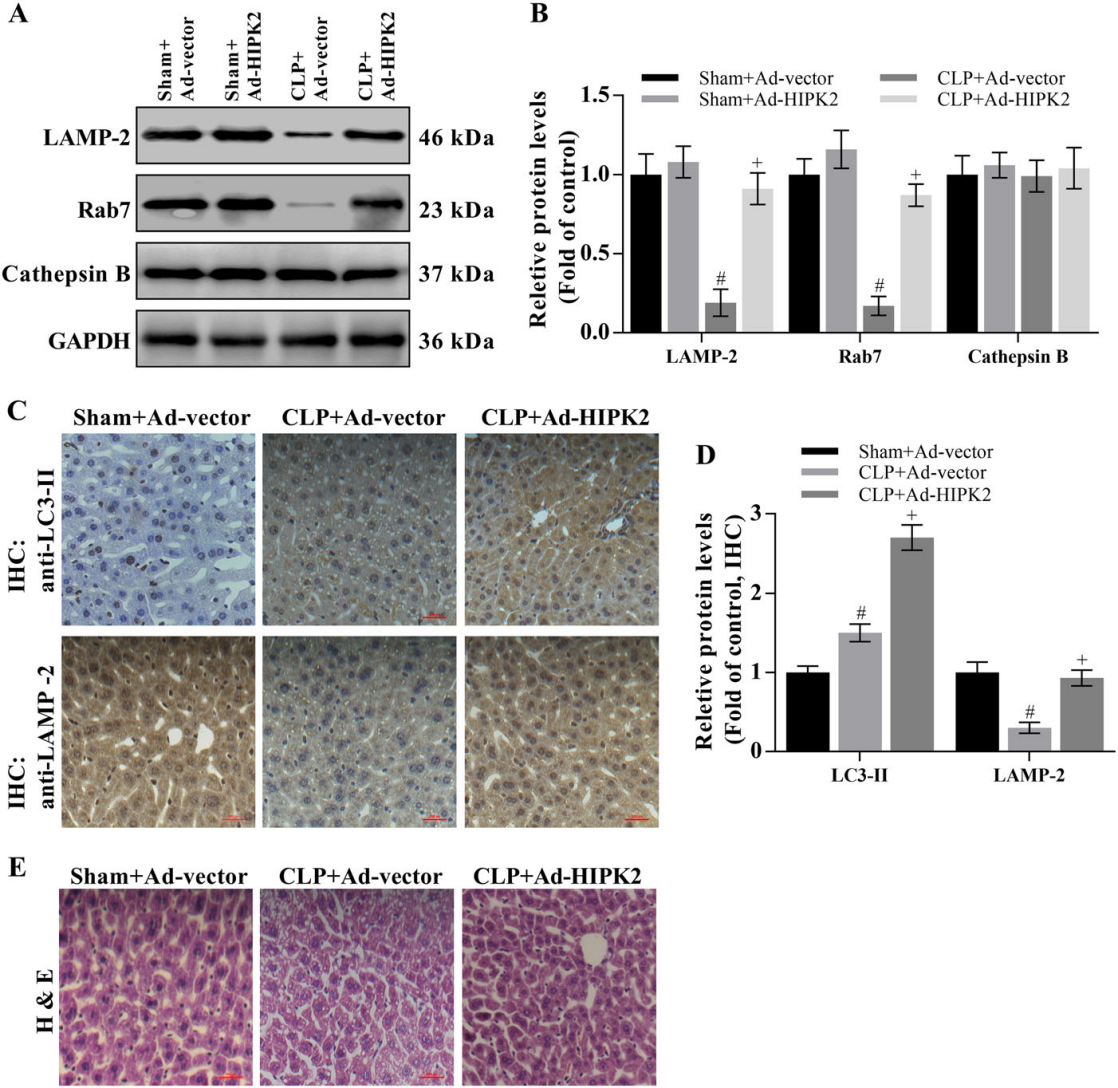
HIPK2 overexpression restores autophagosome–lysosome fusion and degradation in CLP-induced mice. **a**, **b** Effects of Ad-HIPK2 on levels of the LAMP-2, Rab7, and cathepsin B proteins in the liver, as analysed by western blotting (**a**) and the results of the corresponding semi-quantitative analysis of levels of the HIPK2 protein based on the optical density measured using ImageJ software (**b**); data are presented as means ± SEM and are representative of three separate experiments, #*p* < 0.05 compared with the sham plus Ad-vector group (control), + *p* < 0.05 compared with the CLP plus Ad-vector group. **c**, **d** Effects of Ad-HIPK2 on levels of the LC3 and LAMP-2 proteins in the liver, as determined by IHC (**c**) and the results of the corresponding semi-quantitative analysis of levels of the HIPK2 protein based on the optical density measured using ImageJ software (**d**); and data are presented as means ± SEM and are representative of six microscopic fields per group, #*p* < 0.05 compared with the sham plus Ad-vector group (control), + *p* < 0.05 compared with the CLP plus Ad-vector group. **e** Images of H & E-stained liver sections captured at a magnification of 200 × (six microscopic fields per group)

Autophagosomes always fuse with lysosomes, resulting in autolysosome formation, which is required for cargo degradation. This process requires LAMP-2 and Rab7^51–54^. Thus, we determined the levels of LAMP-2, Rab7, and cathepsin B in the mouse liver. Levels of the LAMP-2 and Rab7 proteins were significantly reduced by 73.1% and 76.4%, respectively, compared with those in the control group, and this reduction was reversed by the overexpression of HIPK2 (Fig. 6a, b). However, cathepsin B expression was not altered in any group. To prove that overexpression of HIPK2 may significantly restore sepsis-induced dysregulation of autophagosome–lysosome fusion, the LC3-II and LAMP-2 protein expression levels in the mouse livers were also analysed by immunohistochemistry. Compared with that in the control group, the LC3-II staining was significantly increased and LAMP-2 staining was decreased by CLP (Fig. 6c, d). Interestingly, HIPK2 overexpression enhanced the increase in LC3 expression and reversed the decrease in levels of the LAMP-2 protein (Fig. 6c, d). As evidenced by H & E staining, HIPK2 overexpression exerted a beneficial effect on preserving the hepatocyte shapes in mice with CLP-induced liver injury (Fig. 6e).

### Effect of HIPK2 on CLP-induced mTOR and calpain signalling

**Fig. 7:**
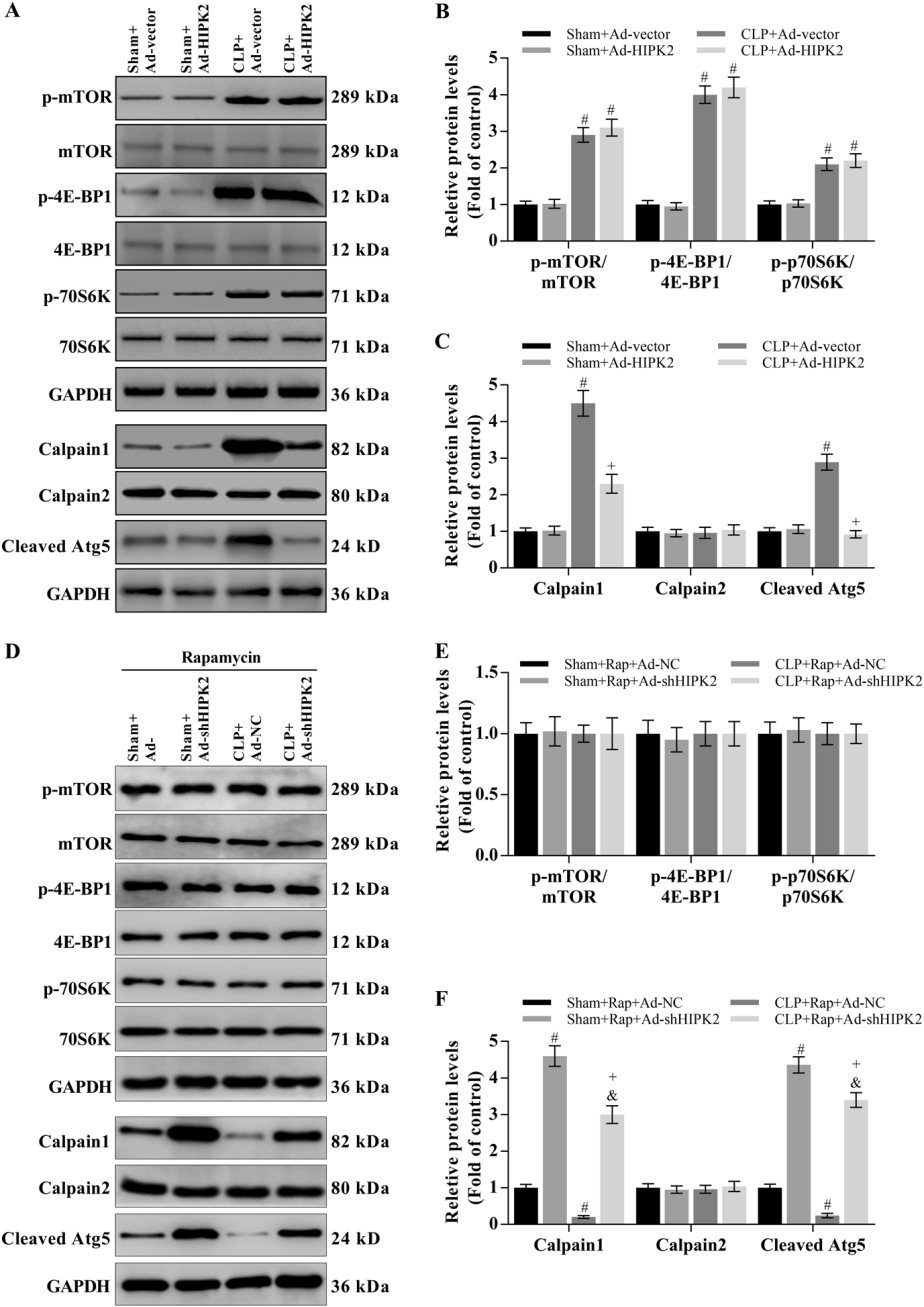
Effect of HIPK2 on CLP-induced mTOR and calpain activation in the mouse liver. **a**, **b**, **c** Effect of Ad-HIPK2 on p-mTOR, p-4E-BP1, and p-p70S6K levels, as determined by western blotting (**a**, **b**) and effect of Ad-HIPK2 on the activation of the calpain system, as analysed by western blotting (**a**, **c**). The results of the corresponding semi-quantitative analysis of protein levels was based on the optical density measured using ImageJ software. Data are presented as means ± SEM and are representative of three separate experiments, #*p* < 0.05 compared with the sham plus Ad-vector group (control), + *p* < 0.05 compared with the CLP plus Ad-vector group. **d**, **e**, **f** Effect of Ad-shHIPK2 on p-mTOR, p-4E-BP1 and p-p70S6K levels after the administration of rapamycin (1 mg/kg per day) for 24 h, as determined by western blotting (**d**, **e**) and effect of Ad-shHIPK2 on the activation of the calpain system in the presence of rapamycin, as analysed by western blotting (**d**, **f**). Results of the corresponding semi-quantitative analysis of protein levels based on the optical density measured using ImageJ software. Data are presented as means ± SEM and are representative of three separate experiments, #*p* < 0.05 compared with the sham plus Ad-NC group, + *p* < 0.05 compared with the CLP plus Ad-NC group, &*p* < 0.05 compared with the sham plus Ad-shHIPK2 group

The calpain system and mTOR signalling play important roles in CLP-induced liver injury^21–25^. We analysed mTOR-mediated signalling and the mTOR-independent pathway (calpain system) to explore the mechanisms by which HIPK2 activates autophagy in response to CLP-induced liver injury. CLP mice exhibited higher levels of phosphorylated mTOR and its downstream targets, 4E-BP1 and p70S6K, than the control group (Fig. 7a, b). The CLP-induced increase in levels of the calpain 1 protein was significantly attenuated by HIPK2 overexpression (Fig. 7a, c). However, no changes in levels of the calpain 2 protein were observed in any group (Fig. 7a, c). Furthermore, CLP mice showed increased levels of the cleaved Atg5 protein (24 kD), which is a downstream target of calpain 1^55^, compared with those in the control group (Fig. 7a, c). However, HIPK2 overexpression attenuated the increase in cleaved Atg5 levels in the livers of CLP-induced mice (Fig. 7a, c).

To investigate the role of mTOR signalling in HIPK2-induced autophagy, we inhibited the activity of mTOR by using rapamycin. HIPK2 knockdown did not change the activation of the mTOR pathway (Fig. 7d, e). However, HIPK2 knockdown significantly increased the levels of calpain 1 and cleaved Atg5 (Fig. 7d, f).

### HIPK2 overexpression reduces elevated cytosolic Ca^2+^ concentrations in LPS-treated primary hepatocytes by interacting with calpain 1 and calmodulin

**Fig. 8:**
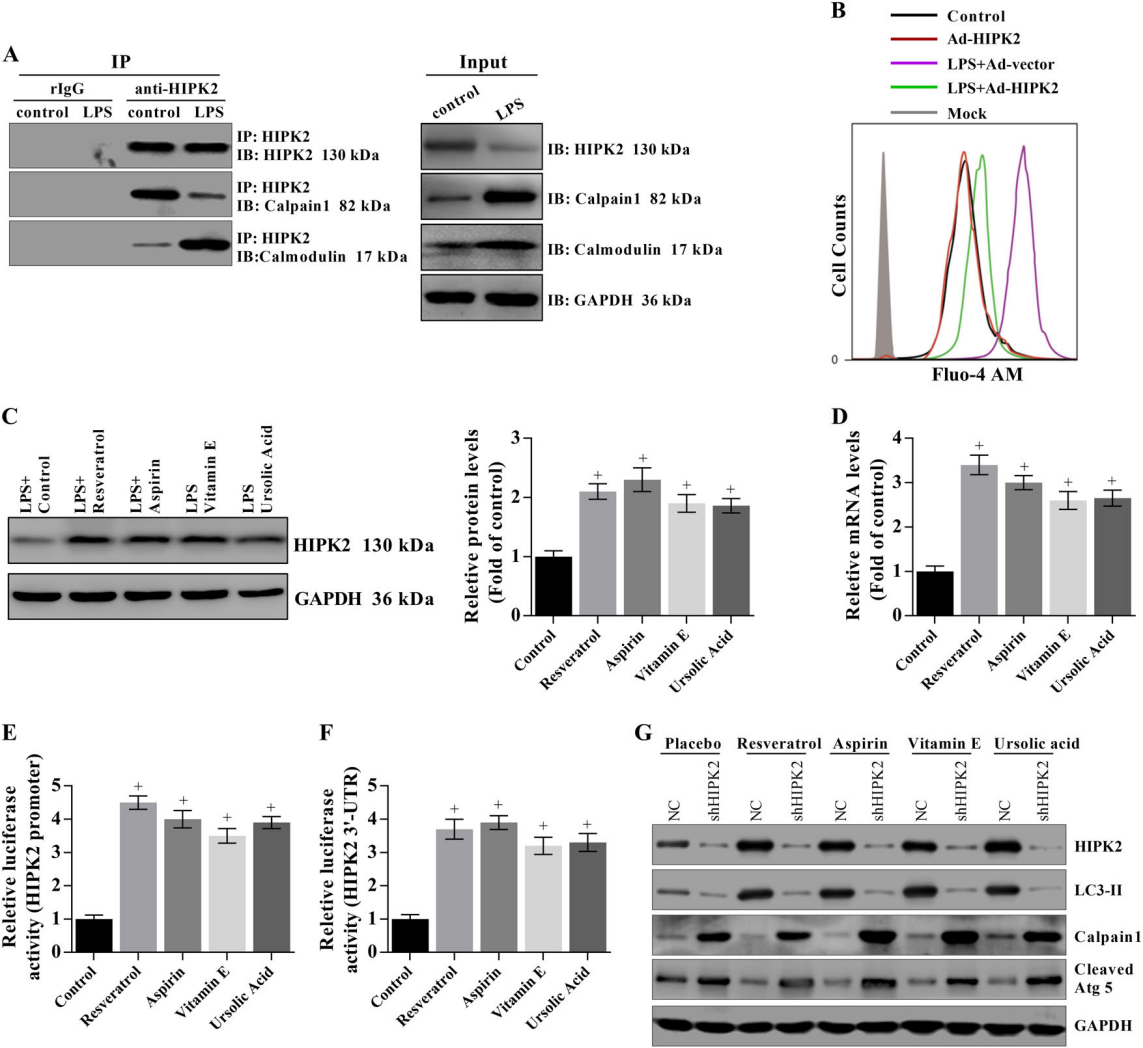
HIPK2 overexpression reduces elevated cytosolic Ca^2+^ concentrations in LPS-treated primary hepatocytes by interacting with calpain 1 or calmodulin. **a** Interactions of HIPK2 with calpain 1 or calmodulin in primary hepatocytes were analysed by immunoprecipitation after an incubation with 0.5 μg/mL LPS for 12 h. **b** HIPK2 overexpression downregulated cytosolic Ca^2+^ concentrations after an incubation with 0.5 μg/mL LPS for 12 h, as determined by flow cytometry. **c**, **d** HIPK2 expression was upregulated by treatments with 5 μM resveratrol, 30 μM aspirin, 10 μM vitamin E, and 15 μM ursolic acid for another 16 h after the LPS treatment, as analysed by western blotting (**c**) and Q-PCR (**d**) and the corresponding semi-quantitative analysis of protein levels was based on the optical density measured using ImageJ software; data are presented as means ± SEM and are representative of three separate experiments, #*p* < 0.05 compared with the LPS plus placebo (control) group. **e**, **f** Effects of the indicated drugs on the luciferase activity of HIPK2 promoter and 3′-UTR stability; the results are presented as means ± SEM and are representative of three separate experiments, #*p* < 0.05 compared with the control. **g** To knockdown HIPK2 expression, the primary hepatocytes were infected with Ad-shHIPK2 (2 × 10^3^ plaque-forming units per well, six-well plate). After 12 h of infection, the effects of the indicated drugs on calpain 1-mediated autophagy were determined by western blotting in three experiments

Calpain is a Ca^2+^-dependent cysteine protease, and calmodulin activity may be regulated by Ca^2+^ to play an important role in cellular signal transduction. Thus, we investigated the interactions of HIPK2 with calpain 1 and calmodulin in LPS-induced hepatocytes. HIPK2 interacted with calpain 1 and calmodulin in hepatocytes (Fig. 8a). However, the interaction of HIPK2 with calpain 1 was weaker in LPS-induced hepatocytes than in the control group and, in contrast, the interaction of HIPK2 and calmodulin was enhanced in LPS-induced hepatocytes compared with that in the control group (Fig. 8a). HIPK2 overexpression significantly decreased the LPS-induced elevation in Ca^2+^ concentrations, as evidenced by flow cytometry (Fig. 8b). Moreover, several anti-inflammatory drugs significantly increased the levels of the HIPK2 protein and mRNA by activating the promoter of the HIPK2 gene and by maintaining the stability of the 3′-UTR, confirming that HIPK2 is a target for sepsis treatments (Fig. 8c–f). Finally, the indicated drugs failed to upregulate LC3-II expression, and they also failed to downregulate calpain 1 expression after HIPK2 knockdown (Fig. 8g).

## Discussion

Autophagy plays an important role in attenuating the sepsis-induced dysfunction of multiple organs and might be an attractive target for the future management of sepsis^16^. Inhibition of autophagy results in increased mortality by causing organ dysfunction and depleting immune cells in patients with sepsis^17,18^. In contrast, upregulation of autophagy inhibits hepatocyte apoptosis and ameliorates liver injury^56,57^. HIPK2 plays a dual role in cancers by either functioning as a tumour suppressor or facilitating tumour progression^27–33^, suggesting that HIPK2 is a positive effector or a negative effector of apoptosis. Moreover, HIPK2 functionally interacts with NRF2^34^, a transcription factor involved in protecting the liver and autophagy^35–40^. Thus, HIPK2 might play an important role in protecting the liver. However, the precise mechanisms by which HIPK2 regulates autophagy and sepsis remain unclear. In this study, HIPK2 overexpression increased the autophagic flux and subsequently increased the survival rate in CLP-induced mice.

Previous studies have demonstrated that autophagy is stimulated in various organs as an early step of sepsis and is characterized by enhanced autophagic vacuole accumulation and the expression of autophagy-associated markers, such as LC3-II, in the livers of CLP-induced animals^56,58^. Activated autophagy exerts a series of beneficial effects by eliminating invading pathogens, inhibiting overproduction of stress-related proteins and improving the function of multiple organelles^59^. LC3-II is only expressed on the mature autophagosomes^56^. SQSTM1/p62, a ubiquitin binding protein, is a robust marker of dynamic autophagic flux, and p62 always directly interacts with LC3-II^56^. Both the LC3-II and p62 proteins are degraded with their cargos in the autolysosome. In the present study, sepsis-induced liver injury was associated with autophagic flux. The hepatic levels of the LC3-II and p62 proteins were significantly increased after CLP. Chloroquine, an autophagy inhibitor, significantly enhanced the increased levels of the LC3-II and p62 proteins. Moreover, chloroquine may enhance liver injury, as evidenced by a lower survival rate. Based on these data, suppression of autophagic flux may be responsible for liver injury in patients with sepsis. Moreover, HIPK2 significantly attenuated liver injury by upregulating LC3-II expression, decreasing levels of the p62 protein, and improving sepsis-induced liver injury that was manifested by decreased levels of ALT, AST, ALP, and inflammatory factors in the liver and serum of CLP mice, leading to the increased survival of CLP mice. Importantly, the HIPK2-mediated restoration of autophagic flux was blocked by chloroquine, and the protective effect of HIPK2 on liver injury was also blocked by chloroquine treatment. Thus, HIPK2 overexpression ameliorated liver injury by restoring the autophagic flux in the livers of mice with sepsis. In addition, NRF2 and NQO1 expression were increased after the overexpression of HIPK2 in septic mice.

The autophagy process involves several steps: initiation and nucleation, expansion, autophagosome formation, autophagosome–lysosome fusion and degradation^60^. Initially, the isolation of membranes from the endomembrane system, such as the Golgi apparatus and endoplasmic reticulum, is initiated by the Beclin-1/Vps34 complex, which is regulated by the ULK complex^17,61–63^. Following nucleation, Atg12-Atg5-conjugated and membrane-associated LC3-II may play important roles in the autophagosome formation^17,61–63^. The Atg12-Atg5 complex always binds to the outer membrane of the phagophore and separates after mature autophagosome formation, which is ultimately completed by the LC3-II protein^17,61–63^. In this process, the Atg3, Atg4, and Atg7 enzymes promote LC3-II production, and autophagosome formation is impaired when Atg3, Atg5, and Atg7 are silenced, according to several studies^17,61–63^. In the present study, HIPK2 overexpression significantly enhanced the increased expression of the Atg12-5 complex and restored the reduced Atg3 expression in mice with sepsis-induced liver injury. HIPK2 overexpression increased the level of the Atg7 protein, which was not affected by CLP-induced sepsis, suggesting that HIPK2 overexpression promotes phagophore expansion and increases mature autophagosome formation in sepsis. In addition, neither HIPK2 overexpression nor sepsis altered the level of the Beclin-1 protein. Thus, HIPK2-mediated autophagy is likely independent of Beclin-1 in sepsis.

The fusion of mature autophagosomes with lysosomes is a key event in the degradation of autophagic cargos^64^. LAMP-1 and LAMP-2 are required for the fusion process^64,65^. When LAMP-2 and Rab7 are downregulated, fusion is impaired and autophagosomes accumulate^51–54^. In the present study, levels of the LAMP-2 and Rab7 proteins were significantly decreased in mice with sepsis-induced liver injury, but levels of the cathepsin B protein were not changed. HIPK2 overexpression ameliorated the decrease in LAMP-2 and Rab7 expression. Furthermore, staining for the LC3-II protein was significantly increased upon overexpression of HIPK2, and LAMP-2 expression was restored by overexpression of HIPK2 in the livers of mice with sepsis. Consequently, HIPK2 overexpression may restore autophagosome–lysosome fusion in mice with sepsis-induced liver injury.

The functions of mTOR have been studied extensively. Under normal conditions, the kinase activity of mTOR is inhibited through a direct interaction with the Atg1-Atg13 complex^66,67^. In the skeletal muscle of mice with sepsis, decreased mTOR activation is associated with reduced protein synthesis^67^. Inhibition of mTOR exerts protective effects on acute kidney injury in mice with endotoxaemia^21^. On the other hand, calpain is a Ca^2+^-dependent cysteine protease that is involved in various biological events, including signalling pathways, apoptosis and autophagy. The activities of calpain 1 and calpain 2, the two main isoforms of calpain, are regulated by micromolar and millimolar Ca^2+^ concentrations, respectively. Therefore, a loss of Ca^2+^ homeostasis affects calpain activity, leading to organ injury^22^. The calpain system suppresses autophagy progression by directly cleaving Atg proteins throughout the autophagy process^23,24^. For example, Atg5 is a specific target of calpains, which often participate in the crosstalk between apoptosis and autophagy^68^. Importantly, the activities and levels of calpain proteins are increased in CLP-induced sepsis models, indicating that calpains inhibit autophagy in sepsis^25^. In the present study, the levels of calpain 1 and cleaved Atg5 and the phosphorylation/activity of mTOR were significantly increased in the livers of mice with sepsis. HIPK2 overexpression reduced the sepsis-mediated increase in calpain 1 and cleaved Atg5 levels but did not affect mTOR levels. Moreover, HIPK2 knockdown significantly increased calpain 1 levels. However, HIPK2 knockdown did not change mTOR signalling when mTOR activity was suppressed by rapamycin. Based on our results, calpain 1 signalling, but not the mTOR pathway, is involved in the HIPK2-mediated increase in autophagic flux. In addition, HPK-1, the sole orthologue of the mammalian HIPK family in *Caenorhabditis elegans*, is required for the formation of autophagosomes and induction of autophagy-related gene expression after nutrient deprivation or mTOR inactivation^69^. Another orthologue of the human HIPK2 gene, Yak1 kinase in *Saccharomyces cerevisiae*, is one such target that is negatively regulated by both the PKA and TOR pathways^70,71^. Thus, HIPK2 and its orthologous proteins (HPK-1 and Yak1) are downstream targets of mTOR.

Moreover, the interaction of HIPK2 with calpain 1 was reduced in mice with sepsis-induced liver injury, and the interaction of HIPK2 with calmodulin was enhanced in septic mice. Calpain 1 activity was monitored by measuring cleaved Atg5 levels^55^. HIPK2 overexpression significantly downregulated the increased Ca^2+^ concentrations in mice with sepsis-induced liver injury. Based on these data, HIPK2 may inhibit calpain 1 activity under normal conditions by interacting with calpain 1, and in response to sepsis, HIPK2 dissociates from calpain 1 and then interacts with calmodulin to decrease Ca^2+^ levels, resulting in attenuation of sepsis-induced liver injury. Resveratrol, aspirin, vitamin E, and ursolic acid have been reported to protect the liver^72–74^. These compounds significantly increased the levels of the HIPK2 mRNA and protein by regulating promoter activity and the stability of the 3′-UTR of the HIPK2 gene, indicating that HIPK2 might be a common and promising target for sepsis treatment. Finally, these drugs failed to downregulate calpain 1 expression after HIPK2 knockdown, implying that HIPK2 is indispensably involved in the protective effects of these drugs.

In conclusion, HIPK2 protected the liver from sepsis-induced injury through calpain 1-mediated autophagy. HIPK2 may inhibit calpain 1 activity and expression under normal conditions by interacting with calpain 1, and in response to sepsis, HIPK2 dissociates from calpain 1 and then interacts with calmodulin to decrease Ca^2+^ levels.

## Electronic supplementary material

Supporting Figure 1

Supplementary figure legends
