# Supplementary figures and images for: Overexpression of homeodomain-interacting protein kinase 2 (HIPK2) attenuates sepsis-mediated liver injury by restoring autophagy
Source: Cell Death Dis. 2018 Aug 28;9(9):847. doi: 10.1038/s41419-018-0838-9 (PMC6113252; doi:10.1038/s41419-018-0838-9)

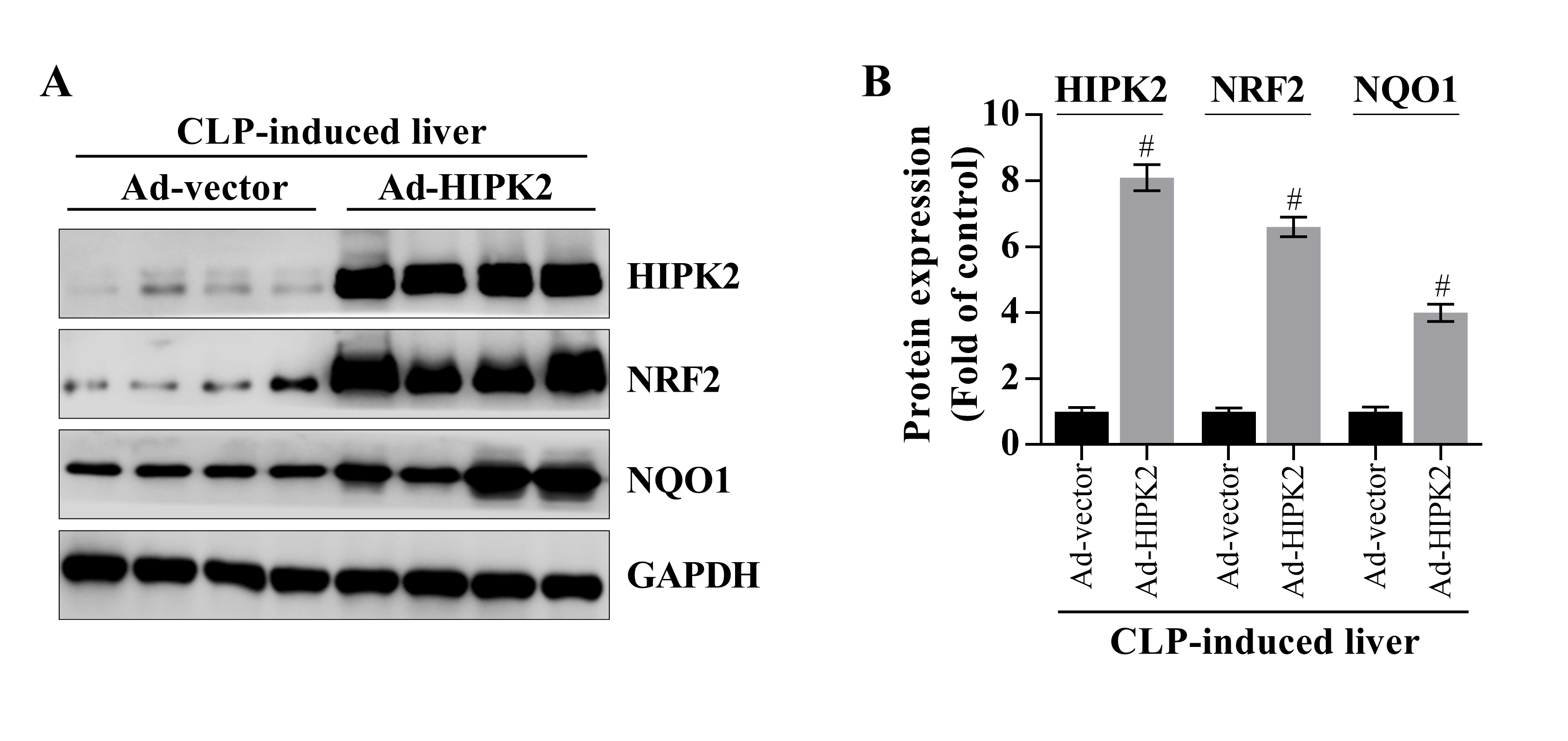

Supplement: Supplementary file 1 — Supporting Figure 1 [file 41419_2018_838_MOESM1_ESM.tif]
