# Supplementary material for: Overexpression of homeodomain-interacting protein kinase 2 (HIPK2) attenuates sepsis-mediated liver injury by restoring autophagy
Source: Cell Death Dis. 2018 Aug 28;9(9):847. doi: 10.1038/s41419-018-0838-9 (PMC6113252; doi:10.1038/s41419-018-0838-9)
Supplement: Supplementary file 2 — Supplementary figure legends [file 41419_2018_838_MOESM2_ESM.docx]

**Supporting Figure 1.** **HIPK2 overexpression increases NRF2 and NQO1 expression.**

(**A**) Western blot analysis of the levels of the indicated proteins. (**B**) The corresponding semi-quantitative analysis of proteins was based on the optical density measured using ImageJ software; the data are presented as means ± SEM and are representative of three separate experiments, #p < 0.05 compared with the sham group.
